# Influence of 3D Microstructure Pattern and Infill Density on the Mechanical and Thermal Properties of PET-G Filaments

**DOI:** 10.3390/polym15102268

**Published:** 2023-05-11

**Authors:** Lucas Lopes, Daniel Reis, Adilson Paula Junior, Manuela Almeida

**Affiliations:** Department of Civil Engineering, ISISE, ARISE, University of Minho, 4804-533 Guimaraes, Portugal; lucas_lopes@civil.uminho.pt (L.L.); daniel.reis@civil.uminho.pt (D.R.); id8639@alunos.uminho.pt (A.P.J.)

**Keywords:** additive manufacturing, thermomechanical testing, fusion deposition modelling, PET-G filaments

## Abstract

This study aims to evaluate the thermal and mechanical performances of PET-G thermoplastics with different 3D microstructure patterns and infill densities. The production costs were also estimated to identify the most cost-effective solution. A total of 12 infill patterns were analysed, including Gyroid, Grid, Hilbert curve, Line, Rectilinear, Stars, Triangles, 3D Honeycomb, Honeycomb, Concentric, Cubic, and Octagram spiral with a fixed infill density of 25%. Different infill densities ranging from 5% to 20% were also tested to determine the best geometries. Thermal tests were conducted in a hotbox test chamber and mechanical properties were evaluated using a series of three-point bending tests. The study used printing parameters to meet the construction sector’s specific needs, including a larger nozzle diameter and printing speed. The internal microstructures led to variations of up to 70% in thermal performance and up to 300% in mechanical performance. For each geometry, the mechanical and thermal performance was highly correlated with the infill pattern, where higher infill improved thermal and mechanical performances. The economic performance showed that, in most cases, except for the Honeycomb and 3D Honeycomb, there were no significant cost differences between infill geometries. These findings can provide valuable insights for selecting the optimal 3D printing parameters in the construction industry.

## 1. Introduction

More than 300 million tons of plastic are produced each year globally, and Europe is the second-largest producer. However, in contrast to the amount produced, only a small percentage is recycled [1], while the rest is incinerated, placed in landfills, or mismanaged. Thus, reducing plastic waste while defining strategies for integrating existing plastic waste into value chains for reuse or recycling and promoting the circular economy is urgently required [2].

Due to the possibilities of additive manufacturing (AM) or 3D printing, it is possible to achieve complex geometries that would be difficult to achieve using conventional construction techniques [3]. 3D printing has been identified as a future direction in the construction sector due to its potential to support sustainable design [4] and can be used to manufacture polymer and recycled polymer-based products [5]. By controlling the internal geometry and different combinations of infill percentages, 3D printing allows for the optimization of thermal conductivity and creates lighter components for use in the energy renovation of buildings [6,7]. Since the building envelope accounts for 50–60% of total heat transference, improving thermal insulation is a cost-effective solution for increasing energy efficiency [8,9].

Most previous studies using polymer filaments for AM focused on mechanical performance as a structural element [6,10] and few studies are available on thermal performance [10,11]. Those studies typically used a smaller extrusion nozzle with a diameter of 0.2–0.4 mm, which may not be ideal in the construction sector due to the long printing time caused by the abundance of raw materials consumed in the sector. To overcome this limitation, using a larger nozzle diameter (e.g., 1.2 mm) and changing printing configurations to increase the printing flow rate allows for increased productivity and buildability. On the downside, it could also reduce the precision of plastic deposition, causing a layered finish that is not desirable in some industries. However, the construction sector often uses finishing elements such as painting, which allows for correcting this type of problem that may occur on the façade. Therefore, higher extrusion nozzles and increased speed printing time are essential to raising productivity [12] and seem compatible with the construction sector. Data evaluating compatibility is certainly highly desirable.

The thermoplastic polyethylene terephthalate glycol (PET-G) is a thermoplastic with good UV resistance, non-toxicity, and broad use in Fusion Deposition Modelling (FDM), which are desired characteristics for a material in a 3D-printed polymer solution for a building renovation scenario. However, other characteristics distinguish the use of PET-G in the construction sector, for example, its transparency. There is research on the use of PET-G as a building façade material due to its UV resistance and transparency [13,14,15,16]. In addition, plenty of additional characteristics can distinguish PET-G in the construction sector. For example, PET-G can be used as a Shape Memory Polymer (SMP), allowing a 4D-printed building solution to be self-assembled and adapted to thermal fluctuations [17,18,19,20]. Although these characteristics are not explored in this study, PET-G additive manufacturing is considered important in construction.

Using the Fusion Deposition Modelling (FDM) method, this study evaluates the thermal and mechanical performances of a set of 3D-printed specimens with a 1.75 mm PET-G thermoplastic filament produced by an industrial 3D printer, Builder Extreme 1500 Pro, Amsterdam, Netherlands, using a 1.2 mm nozzle and a 0.6-mm-layer height. Different configurations of core topologies and infill percentages were tested in the laboratory to investigate thermal resistance variations and tensile strength (MPa). Based on the results, the microarchitecture of the 3D printing configuration was investigated with the aim of achieving an optimized design for thermal resistance without compromising mechanical strength. The experiment was carried out at high print speeds and a 1.2 mm nozzle—above the 0.4 mm used in the current literature—was used to manufacture building components for energy renovation of building façades.

## 2. Materials and Methods

### 2.1. Printing Configurations and Materials

All the samples were printed using the industrial 3D printer model Builder Extreme 1500 Pro with 1.75 mm polyethylene terephthalate glycol (PET-G) filament in the colour glacier white by the company Winkle. The CAD models were developed using the Sharp3D software due to their availability and ability to export 3 MF and STL files. The chosen slicing software for exporting the g-code was PrusaSlicer, version 2.4.0, due to its internal library of 3D geometries. The printing settings used are listed in Table 1. Additional values, such as retraction and printer head acceleration, were defined in previous tests, ensuring minimal stringing, ghosting, and other printing defects.

The polymer PET-G was selected due to its low thermal conductivity, hygrothermal performance, UV radiation resistance, and good mechanical strength compared with other available materials [17,18]; these are some of the performance criteria generally required for energy renovation of façade panels. However, other factors that make PET-G a suitable material for various uses, such as its biocompatibility and transparency, were deemed less important to the research goals. Furthermore, other polymers with these characteristics, such as PVC, can be used to create a building insulation panel. Additionally, PET-G is a filament that is already used in the construction sector, facilitating comparison with other studies [14,15,21]. Other polymers that are also commonly used in 3D printing, such as PLA and ABS, were discarded as options due to their inadequacy in meeting the requirements for a building insulation panel, such as resistance to UV or waterproofing.

**Figure 1:**
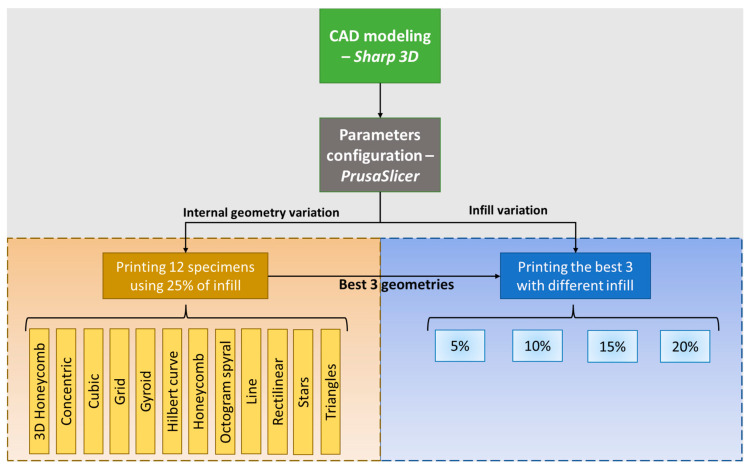
Schematic of the methodology adopted in this study. First, the tests were conducted for the 25% of infills for 12 geometries. Then, different infill densities (5–20%) were used to print new samples for the three best thermal geometries for thermal and mechanical tests.

The experiment was divided into twelve geometries to quantify their influence on thermal performance, and twenty-four to test the mechanical performance—twelve in each direction (X and Y directions). A fixed infill of 25% was used for thermal and mechanical performance evaluation. Based on the results, the three geometries with the best thermal performances at different densities—5%, 10%, 15%, and 20%—were printed, giving a total of 12 samples for analysis of thermal performance and 24 samples for analysis of mechanical performance. Figure 1 illustrates the process of specimen preparation adopted in this study. The infill geometry patterns tested were selected due to their availability in various commercial slicing software. The following microarchitecture configurations were tested: 3D Honeycomb, Concentric, Cubic, Grid, Gyroid, Hilbert curve, Honeycomb, Octagram spiral, Rectilinear, Stars, and Triangles. Since the goal of this study was to optimize thermal performance without compromising mechanical resistance, lower densities, between 5% and 25%, were evaluated since the thermal conductivity of air (0.025 W.m^−1^.°C^−1^) is lower than that of the thermoplastics (around 0.20 W.m^−1^.°C^−1^) [22].

### 2.2. Thermal Tests

The 3D-printed samples were thermally tested in a hotbox test chamber located at the Physics and Technology Laboratory of the Department of Civil Engineering of the University of Minho. It was built following ASTM C1363-11:2011 [23] and validated by Teixeira and colleagues [24]. It consists of two five-sided chambers (hot and cold) with 1 m^3^ of internal volume each connected by a central assembly ring where the tested specimens are placed. The envelope is well insulated and made of extruded polystyrene (20 cm thick, thermal conductivity (λ) = 0.037 W/(m.K) to reduce heat flow through the envelope and minimize conductive heat losses.

On the back wall of the cold chamber, two ventilation devices, which allow the balance of the air temperature in the chamber, are positioned to maintain uniform heat flow through the sample. In the hot chamber is a heating system whose temperature is limited to a predefined range (32 ± 2 °C).

**Figure 2:**
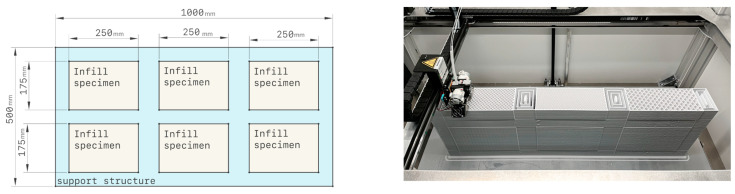
Panel sizes and configurations used for the thermal tests (**left**) and the printing setup (**right**), with an overall size of 1000 × 500 × 100 mm.

The tests were carried out on 1000 × 500 × 100 mm panels and the size of each specimen was 175 × 250 × 100 mm, as shown in Figure 2. The panels were positioned on the mounting ring between the two hotbox chambers to determine each sample’s thermal resistance (R-value) using the heat flow method, based on the methodology in ISO 9869-1:2014 [25].

The experiment used green TEG equipment, positioning the sensors in the centre of the samples to quantify the temperatures on both sides and the heat flux through the material. Based on the manufacturer’s data, the accuracy of the temperature-measuring devices is ±0.1 K and the accuracy of heat flow measurement is ±3%. The R-Value is calculated from Equation (1).

(1) $$R=\frac{\sum_{j=1}^{n}\left(T_{sij}-T_{sej}\right)}{\sum_{j=1}^{n}q_{j}}$$

where

*q_j_* is the heat flux at time *j*;
*T_sij_* is the inside surface temperature at time *j*;
*T_sej_* is the outside surface temperature at time *j*.

The thermal conductivity value is obtained from the ratio between the thickness of the tested sample and the thermal resistance obtained. The thermal tests were validated by simultaneously performing tests on a material whose thermal conductivity was already known: an XPS plate, where a value of 0.030 W/m.K was obtained within the range 0.029–0.036 W/m.K presented by the technical dossier from the Portuguese Danosa manufacturer’s technical report [26] for this type of material.

**Figure 3:**
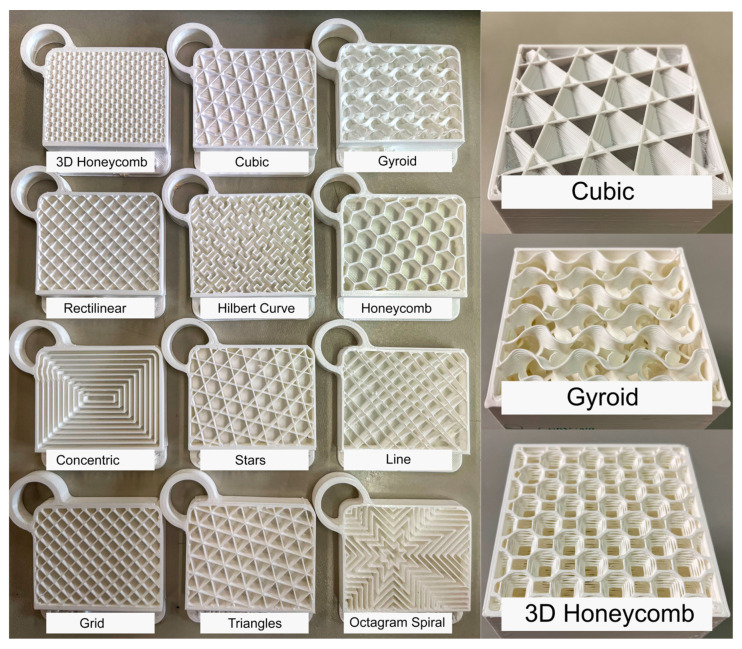
Test panel section views displaying the 12 infill geometries tested and the three-dimensional structures.

The panels were printed with six internal geometry variations, totalizing 12 samples, as illustrated in Figure 3. Subsequently, the three best internal geometries were again subjected to thermal tests to identify the best infill patterns based on thermal performance.

### 2.3. Mechanical Tests

The flexure resistance indicates the load capacity of different plastic materials and core structures [27]. Therefore, the bending behaviour of the 3D-printed samples was carried out following the standard ASTM C393 [28]. The standard configuration was used considering a distance of 150 mm between the support bars and a speed test for a crosshead displacement of 6 mm/min [28]. A steel sheet was placed in the middle of each sample to distribute the bending force over the sample. The three-point bending test was carried out using a universal material testing machine, MTS Exceed E45, with a 1000 Hz data acquisition rate, to ensure accurate results. The experiments were conducted at room temperature and the failure force (kN) was quantified. The samples were modelled using the Sharp3D software and printing parameters of the PrusaSlicer software were used, similar to the thermal tests.

**Figure 4:**
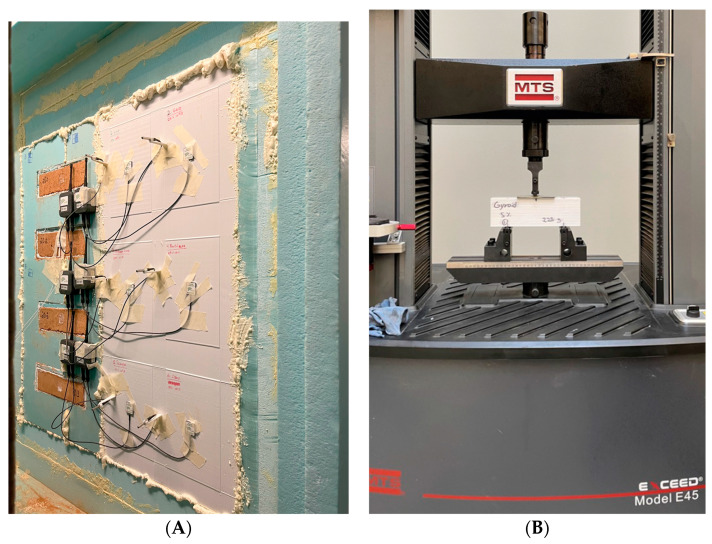
Left (**A**) is the thermal test procedure using a hotbox. Right (**B**) is the bending testing procedure in an MTS Exceed E45. Photo taken by the authors during the experimental tests.

Depending on where the load is applied (horizontal or vertical), the tensile strength will vary due to the geometric effect. For this reason, the tests included two directions—X and Y orientations. In addition, five specimens were evaluated per test condition to improve the reliability of the results. This procedure is also suggested in the ASTM C393 standard [28]. A total of 80 samples were printed, including Stars and Honeycomb geometries, four infill patterns (5%, 10%, 15%, 20%), two different orientations, and five samples of each (2 × 4 × 2 × 5 = 80 samples). The equipment used to run the mechanical bending test was an MST Exceed Model E45. Figure 4A and Figure 4B illustrates the procedure used to run the thermal tests and the bending test, respectively.

### 2.4. Operational Cost Analyses

The estimation of material and energy costs considered the estimated cost differences between the geometries; these are printing time and material costs. To define the 3D-printing costs, the market price of the filament used was 23.00 €/Kg and the energy cost of electricity was 0.1833 €/kWh based on the average European price for commercial energy [29].

Due to geometry configurations, there are variations in the amount of filament used to anchor the infill to the external perimeter of the 3D-printed object. Therefore, these variations will result in the consumption of different quantities of materials based on the infill geometry. The estimated weight of the 3D-printed object was based on the slicing software. To ensure comparability of the results, the same software and slicing were used to analyse material consumption for all the infill geometries, the PrusaSlicer. Subsequently, the weight of each infill was converted to the price of the filament weight.

The energy cost was calculated taking into account the variations in the printing times of each infill geometry. Next, the equipment cost was calculated based on 300 Wh 3D-printing energy consumption. Finally, the printing time was estimated using the PrusaSlicer slicing software.

The simulation in PrusaSlicer was done with the configuration of the Builder Extreme Pro 1500 3D Printer, simulating the print of a 100 cm × 50 cm × 10 cm panel. The weight and print time values were then converted to print a 1 m^3^ cube. The results of the estimated cost of each infill were used to rank the infills in terms of costs. The results were later compared to the mechanical and thermal performance costs.

### 2.5. Combined Information Analysis

The results of the thermal, mechanical, and cost experiments were combined through a normalization process. The analysis considered the 12 infill geometries with 25% infill density, as it was a common configuration in all previous experiments. The optimization procedure used to generate the graph was an adaptation of the Mixtri 2.0 software for Microsoft Excel [30], which was used to find an optimized geometry based on the experiments conducted. The adaptation replaced the categories considered in the original software with thermal performance, mechanical performance, and costs. As lower cost values are preferred for the 3D-printed infill geometries, its normalized value was inverted in the software, thus 0.00 represented the best value and 1.00 represented the worst value.

## 3. Results and Discussion

### 3.1. Geometric Effect

#### 3.1.1. Mechanical Performance

**Figure 5:**
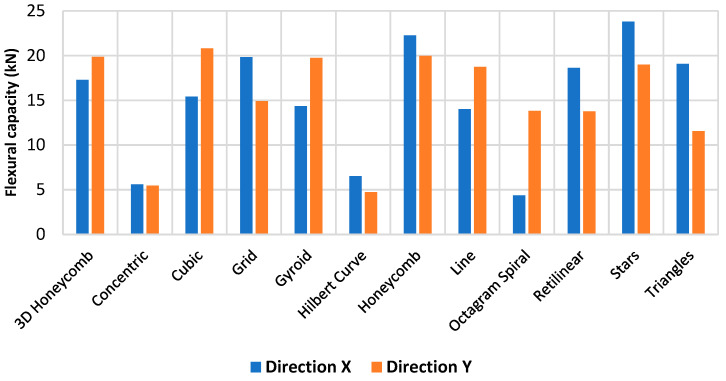
Three-point bending tests for 12 geometries using a fixed infill density of 25%. Two directions were tested.

The three-bending tests revealed that structural geometries play an important role in the bending behaviour, as illustrated in Figure 5, varying between 5 and 20 kN using a fixing infill density of 25%. Furthermore, due to the anisotropic nature of the geometries, the performance varies between orientations.

Concentric and Hilbert Curve have the lowest performance, with a failure force of around 5 kN in both X and Y directions. The difference can be explained by the low surface contact of the internal geometry with an outer perimeter of the sample, resulting in lower structural support. Honeycombs have the best performance and the lowest variation between directions since they have more filaments between cell walls. Honeycomb also showed good bending performance in the literature [27].

**Figure 6:**
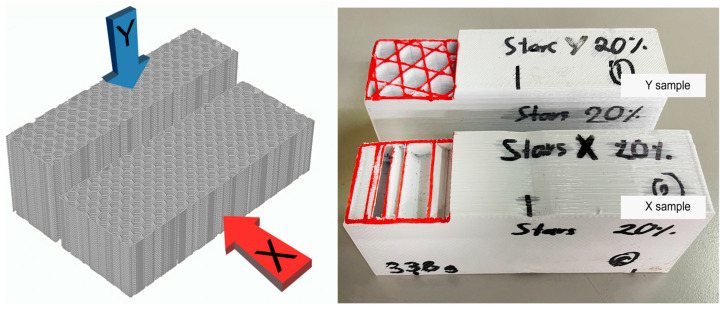
X and Y samples for mechanical performance experiments, where X was tested parallel to the printing plane, and Y was tested perpendicular to the printing plane.

The variations in mechanical performances observed in the X and Y directions can be attributed to the printing layer orientation and the geometry of the infill. As shown in Figure 6, sample X was tested parallel to the printing layer plane, while sample Y was tested perpendicular to the printing layer plane. The infills 3D Honeycomb, Cubic, Gyroid, Line, and Octagram Spiral gave better results when tested in the Y direction, while the geometries Concentric, Grid, Hilbert Curve, Honeycomb, Rectilinear, Stars, and Triangles performed better in the X direction. Since the tests samples had an external perimeter thickness of 1.2 mm, the geometries Concentric and Hilbert Curve in the X and Y samples, and Octagram Spiral in the X samples, mechanical performance can be attributed to the resistance of the external perimeters more than the infill.

For the geometries with the best thermal and mechanical results—Gyroid, Stars, and Honeycomb—a set of samples with different densities—5%, 10%, 15%, and 20%—were printed. The results are presented in the Section 3.2.

#### 3.1.2. Thermal Performance

**Figure 7:**
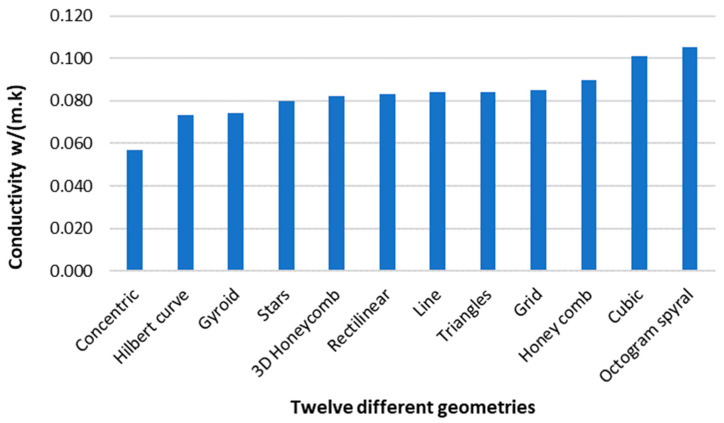
Conductivities (W/m.K) of twelve different geometries, with a fixed density of 25%, were tested in a hotbox.

The influence of the internal microarchitecture on thermal conductivity W/(m.K) is presented in Figure 7. The density was constant for each sample at 25% (i.e., 307 kg/m^3^).

With a thermal conductivity of 0.057 W/(m.K), the Concentric geometry gave the best result among the tested geometries since it made no direct contact with the external perimeter. Although the Concentric geometry had good thermal performance, this geometry offers no structural support to the external perimeters, which may result in a specimen unsuitable in applications where the 3D-printed insulation may be exposed to impact. The second-best materials in terms of thermal insulation were the Hilbert curve and Gyroid, with 0.074 W/(m.K). Usually, a material can be considered a thermal insulator if its conductivity is lower than 0.070 W/(m.K) [31]. Only the Concentric geometry met this requirement.

The few studies that have evaluated the influence of 3D-printed internal geometries on thermal performance have reported similar results. The study [32] gave a thermal conductivity of 0.037 W/(m.K), using a porous structure made with PLA, and a density of 366 kg/m^3^. Another study [33] evaluated the thermal conductivity of 3D-printed plastics using the stereolithography (SLA) method to analyse different sizes, densities, and infill patterns—rectilinear, Honeycomb, and triangular. The lowest thermal conductivity was obtained for Honeycomb and rectilinear, with 0.0591 W/(m.K) at a density of 180 kg/m^3^. This result is lower than that obtained in this study—0.083 W/(m.K) for rectilinear and 0.090 W/(m.K) for Honeycomb, since the density is 40% lower. The result can be justified by the lower thermal conductivity of air (0.025 W/(m.K)) compared with that of thermoplastics (around 0.20 W/(m.K)) [22]. In theory, increasing the air voids can lead to better thermal performance. However, since the density is lower, the mechanical performance is also reduced as a trade-off [34].

In the next section, the effects of infill percentages on thermal performance are evaluated for the three best geometries.

### 3.2. Infill Pattern Effect

#### 3.2.1. Mechanical Performance

**Figure 8:**
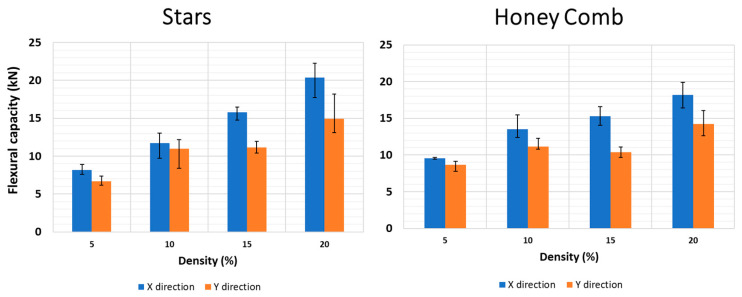
Three-point bending test results for Stars (**left**) and Honeycomb (**right**) with different densities (5–20%) and two directions (X and Y directions). The uncertainty range represents the maximum and minimum values obtained in the tests.

The effect of infill on the bending performance is illustrated in Figure 8 for the Stars and Honeycomb geometries. As expected, the variations in infill densities (%) and mechanical performances follow the same trend, with increased weight increasing the bending performance (kN). A similar trend is suggested in the literature [35]. The difference in the mechanical performance between the X and Y samples is explained by the geometries of the stars and honeycombs, where the X samples have the bending force applied parallel to the printed layer.

The uncertainty range is included and represents the minimum and maximum values obtained from the mechanical test of each infill density (%). This variation is due to the technological limitation of the 3D printer used to print the samples, resulting in a variation range between 3% and 28% for the minimum and maximum values. Despite being an industrial 3D printer model, some precision limitations still influence the mechanical flexure results.

This variation is mainly due to the differences found in the weights of samples, varying between 0% and 9%, for each set of five samples, considering the same density and the same X and Y directions. The higher the variation in weights between the samples, the greater the margin of uncertainty in the bending tests.

**Figure 9:**
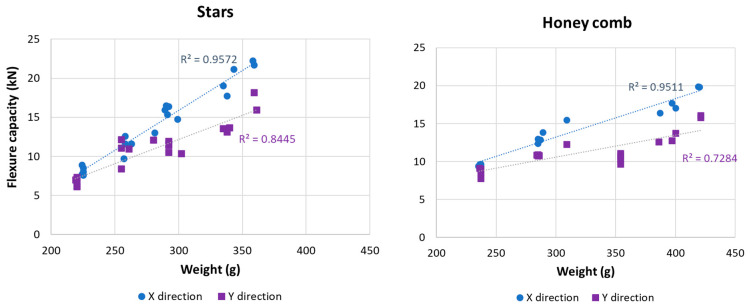
Correlations between the weight (grams) and three-point bending performance (kN) of Stars geometry (**left**) and Honeycomb geometry (**right**).

Additionally, and for the same weight, a small variation in the flexure capacity was found, as illustrated in Figure 9. For example, in Stars geometry in the Y direction, the flexure capacity for 250 g varies between 8 kN and 12 kN. Despite this variation, the results provide a good linear correlation between weight and mechanical performance, with a linear correlation of 0.95 and 0.73–0.85 for the X and Y directions, respectively. This variation is due to 3D printer limitations and the absence of a controlled temperature in the printing chamber since the printing of the samples occurred consecutively over a few days and nights, resulting in variations in the environmental temperature.

In the literature, the study [27] included a series of three-point bending tests performed using 30% infill density and found a variation of 0.2–0.8 kN, which depended on the geometry and material used (ABS and ASA). The results obtained are an order of magnitude lower than those obtained in this study. The lower magnitude is attributed to the study [27], which used a layer 0.2 mm thick, whereas the current study used 0.60 mm, resulting in more material in the core structure and thus better mechanical performance. Therefore, the results suggest that the configuration used to model the samples can produce high-resistance thermal panels for building façade.

#### 3.2.2. Thermal Performance

**Figure 10:**
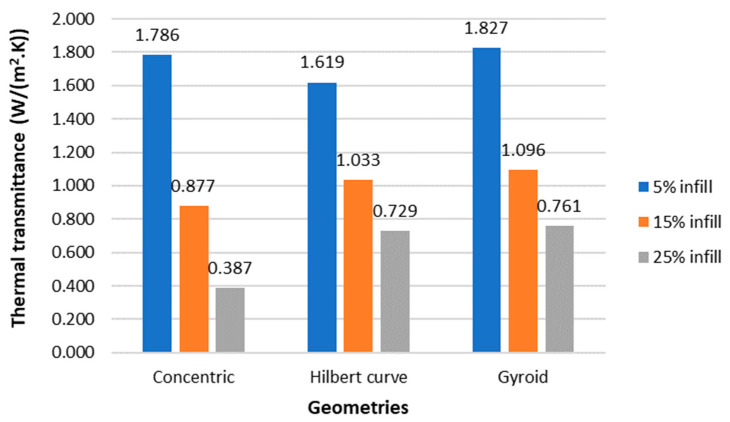
Thermal transmittance of the three geometries that presented the best thermal results printed with different densities (5, 15, and 25%).

Figure 10 shows the effect of infill on the thermal performance of the internal geometries that gave the highest insulation potential (Concentric, Hilbert curve, and Gyroid). The thermal transmittance (U-value) results were presented and calculated from the R-values obtained for each specimen. As can be seen, the results obtained from the attempt to optimize the infill geometries were counterintuitive, going against the premise that lower-density infills would allow greater air entrapment, making the solution more thermally resistant.

**Figure 11:**
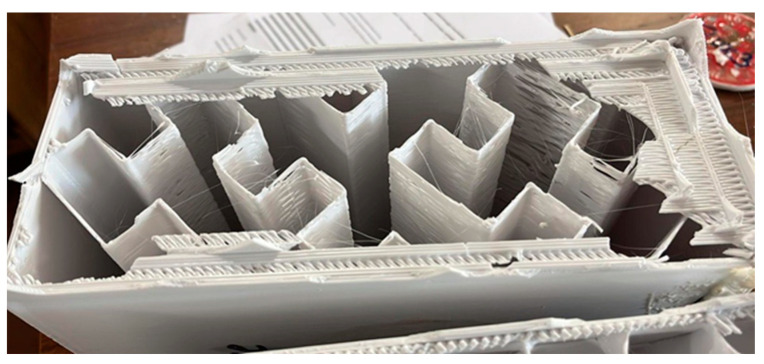
Cross-section of the Hilbert curve specimen with 5% infill density.

Based on Figure 10, the specimens with a 5% density had the worst thermal results. This phenomenon may mainly be associated with possible printing failures arising at lower percentage densities that affect the U-value, as seen in Figure 11, which shows the post-test state of the Hilbert curve geometry specimen with 5% density. Analogous behaviour was also identified in the study [36] using a robotic polymer extruder, where thermal transmittance (U-value) varied from 1.7 to 1.0 W/(m^2^.K) only by changing the infill density. The best performance was obtained using higher infill densities, which is consistent with the values obtained in this study, where the U-values vary between 1.7 and 1.8 W/(m^2^.K) for 5% infill density and 0.4–0.8 W/(m^2^.K) for 25% infill density. Another study [11] used PLA filaments to print a 3D block with low infill density for thermal insulation. The authors obtained a 1.2–1.4 W/(m^2^.K) U-value [11].

According to Portuguese regulations [37], the external wall’s U-value must be below 0.35–0.50, depending on the climate zone. Based on this study’s data, the Concentric geometry with 25% infill density has a U-value of 0.387 W/(m^2^.K), which complies with the Portuguese regulations and can therefore be used as an insulation material and, at the same time, has good mechanical performance.

On the other hand, from a resource use efficiency point of view, it would be better to reduce the infill density in order to consume less material. A possible solution could be combining low infill density, by using the concentric geometry, with other thermal insulation materials. In the literature, good results were obtained using 3D-printed opaque blocks filled with polystyrene and wool, improving thermal transmittance by 76–215% compared with blocks with only air cavities [12]. Further research should be conducted to evaluate this possibility and to test it using different natural insulation materials, including renewable natural materials such as wood fibres, mineral wool, and straw [38].

### 3.3. Material Costs

The costs of the materials are shown in Table 2. The printed samples had similar values, around 335 Kg/m^3^ for the 25% infill, 206 Kg/m^3^ for the 15% infill, and 77 Kg/m^3^ for the 5% infill. Although the results are similar, the Honeycomb and 3D Honeycomb geometries displayed significantly higher material consumption and printing times. These higher values in material consumption can be attributed to the higher anchorage points required for these geometries. At the same time, the high printing time values are associated with the acceleration and deceleration of the printing head during the manufacturing process. The geometries with continuous printing movements, such as Grid, Line, Rectilinear, Stars, Triangles, and Gyroid, had the lowest printing times and, therefore, lower energy consumption. However, due to the relatively low energy consumption associated with the 3D-printing process, the energy consumed during printing is not as relevant as the cost of the material consumed. On an industrial scale, the cost of the printing material could be significantly lower and therefore give more feasible values.

**Figure 12:**
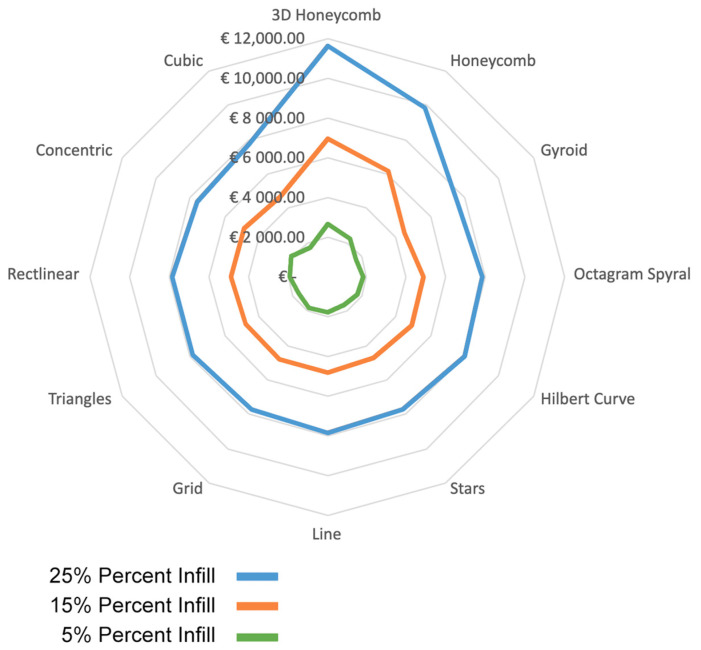
Material costs for the different geometries and infill densities.

When comparing the general costs of the internal geometries, it was observed that although the cost decreased significantly with the decrease in infill density, the geometry cost ranking did not change significantly. Nevertheless, a reduction of up to 75% in costs and up to 77% in weight was observed. The 3D Honeycomb and Honeycomb geometries had considerably higher costs compared with the other geometries. Meanwhile, Gyroid had the lowest infill material consumption, and therefore, was the best in terms of costs, even though it was not among the geometries with the lowest printing times, as shown in Figure 12.

### 3.4. Combined Information

The combined information between the thermal and mechanical performances was normalized to the costs of the infill geometries to understand how the infill geometries can be optimized based on the combined values of the results obtained. The normalized values are shown in Table 3.

The normalized values can be used, to a limited degree, to compare results between infill geometries. However, we observed that infill geometry optimization varies based on the weighted value of each aspect.

**Figure 13:**
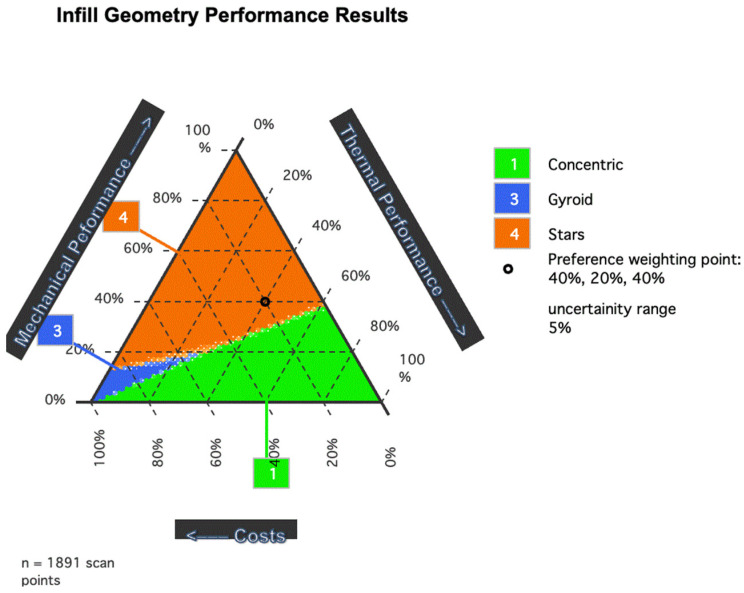
Optimization graph of the combined values of the results obtained from the mechanical, thermal, and costs associated with the 25% infill density geometries.

Figure 13 shows the optimization results for the normalized values. The optimization results revealed the three optimal infill geometries as Stars, Concentric, and Gyroid. The Stars infill gives the best-combined performance value for most weight variations. However, in cases where the mechanical performance is a small percentage of the weight, the Concentric and Gyroid infills are optimal, with the Gyroid having a lower percentage. When thermal performance is valued, with no importance given to mechanical performance, the Concentric infill geometry is shown to be the optimal infill geometry configuration. When the main concern is the cost of the solution, Gyroid is the optimal infill in this study scenario.

## 4. Conclusions

When comparing thermal performance, mechanical performance, and costs of the above commercially available infill geometries, there was no single infill geometry and configuration that is optimal for every scenario. However, it was observed that even these commercial infill geometries can significantly impact the costs and performance of a 3D-printed object, even that targeted to the construction sector. To better use and adapt the 3D-printed infill to each case, geometric performances should be weighed based on the importance of their use. An optimal infill for specific applications can be designed with proper research. For example, in a 3D-printed building renovation panel, the optimal infill should balance thermal and mechanical performance. Although an optimal infill was identified based on the limited experiments in this research, there is plenty of research required to obtain an accurate optimal infill geometry.

Thermal performance analysis concluded that there is a significant difference between the different internal geometries. However, when analysing the impact of decreasing infill density, a decrease in thermal performance was also observed. Further thermal performance improvements can be achieved by stuffing the panel with other insulation materials [12]. Therefore, further development of an optimized infill density and geometry could focus on integrating insulation materials into the 3D-printed object.

The mechanical performance of the 3D-printed object varied greatly based on the infill geometry and density. The best infill configuration for mechanical performance was four times better than the lowest-performing geometry. Moreover, the mechanical performance tests showed a significant variation in performance based on the printing orientation; direction X showed better overall results in the highest-performing samples. A significant drop in performance was observed due to the decrease in infill density. Even though the samples with 5% infill gave a lower score, it was observed that the samples had relatively good performance when the external perimeter was 1.2 mm. Overall, the results have shown that infill geometry is essential to a 3D-printed object and that infill density is correlated with mechanical performance. Further research could be conducted to optimize the flow rate of the polymer during 3D printing or with 4D printing programming to optimize the object’s mechanical performance to the desired requirements.

The economic performance showed that in most cases—except for the Honeycomb and 3D Honeycomb—there is no significant cost difference between infill geometries. Complex geometries with several acceleration and deceleration points, such as Honeycomb, 3D Honeycomb, and Hilbert Curve, had significantly higher printing times and material consumption. However, the general cost differences were less significant than the differences in thermal and mechanical performances.

The overall characteristics of the printing and the results showed that good thermal and mechanical performance results can be achieved even when exclusively using 3D printing of the internal geometry. Therefore, the infill shows promising development capacity for the proposed use and further research involving the use of the internal geometry as an insulation solution for building energy renovation will be conducted.

## Figures and Tables

**Table 1:** 3D Printing configurations used to print samples for the thermal and mechanical tests.

Parameters	Adopted Values
Nozzle diameter	1.2 mm
Printing temperature	250 °C
Printing bed temperature	60 °C
Layer height	0.60 mm
First layer height	0.45 mm
Extrusion width	1.26 mm
First layer print speed	45 mm/s
Print speed	70 mm/s

**Table 2:** Infill geometry and density cost, printing time, and material per cubic meter.

Density and Geometry	Weight (kg/m^3^)	Printing Time (h/m^3^)	Cost (€/m^3^)
25% Gyroid	325.0	1495	7556.74 €/m^3^
15% Gyroid	193.2	651	4479.38 €/m^3^
5% Gyroid	73.8	212	1708.21 €/m^3^
25% Concentric	329.1	735	7608.70 €/m^3^
15% Concentric	209.4	470	4842.02 €/m^3^
5% Concentric	89.6	204	2072.01 €/m^3^
25% Grid	332.7	846	7698.05 €/m^3^
15% Grid	206.0	528	4767.95 €/m^3^
5% Grid	79.6	207	1842.16 €/m^3^
25% Stars	333.0	886	7706.95 €/m^3^
15% Stars	202.7	549	4691.35 €/m^3^
5% Stars	72.3	212	1673.97 €/m^3^
25% Cubic	335.1	919	7757.51 €/m^3^
15% Cubic	204.2	566	4727.29 €/m^3^
5% Cubic	72.5	209	1679.89 €/m^3^
25% Triangles	337.3	967	7811.71 €/m^3^
15% Triangles	205.1	589	4749.21 €/m^3^
5% Triangles	72.7	218	1684.55 €/m^3^
25% Line	339.8	864	7862.86 €/m^3^
15% Line	209.1	537	4839.27 €/m^3^
5% Line	78.6	203	1818.48 €/m^3^
25% Rectilinear	339.3	870	7866.51 €/m^3^
15% Rectilinear	210.5	546	4870.60 €/m^3^
5% Rectilinear	81.0	210	1874.55 €/m^3^
25% Octagram Spiral	337.3	1024	7814.27 €/m^3^
15% Octagram Spiral	208.3	633	4825.23 €/m^3^
5% Octagram Spiral	77.9	253	1805.15 €/m^3^
25% Hilbert Curve	344.1	2146	8033.45 €/m^3^
15% Hilbert Curve	213.0	1215	4965.81 €/m^3^
5% Hilbert Curve	75.9	332	1763.93 €/m^3^
25% Honeycomb	419.4	2969	9809.84 €/m^3^
15% Honeycomb	263.5	1554	6146.90 €/m^3^
5% Honeycomb	95.8	378	2225.13 €/m^3^
25% 3D Honeycomb	498.8	2868	11,630.99 €/m^3^
15% 3D Honeycomb	298.1	1488	6937.64 €/m^3^
5% 3D Honeycomb	113.9	379	2641.48 €/m^3^

**Table 3:** Normalized values of the infill geometry performances.

Infill Geometry	Mechanical Performance	Costs	Thermal Performance
Concentric	1.00	0.01	1.00
Hilbert Curve	0.99	0.12	0.51
Gyroid	0.28	0.00	0.50
Stars	0.00	0.04	0.38
3D Honeycomb	0.18	1.00	0.33
Rectilinear	0.33	0.08	0.32
Line	0.32	0.08	0.30
Triangles	0.38	0.06	0.30
Grid	0.25	0.03	0.28
Honeycomb	0.02	0.55	0.21
Cubic	0.22	0.05	0.05
Octagram Spiral	0.78	0.06	0.00

## Data Availability

All the necessary data is given in the article.
